# Synergistic Antibacterial Activity of the Essential Oil of Aguaribay (*Schinus molle* L.)

**DOI:** 10.3390/molecules171012023

**Published:** 2012-10-12

**Authors:** Pedro M. de Mendonça Rocha, Jesus M. Rodilla, David Díez, Heriberto Elder, Maria Silvia Guala, Lúcia A. Silva, Eunice Baltazar Pombo

**Affiliations:** 1 Departamento de Química, Unidade I&D Materiais Têxteis e do Papel, Universidade da Beira Interior, 6201-001 Covilhã, Portugal; 2 Departamento de Química Orgánica, Universidad de Salamanca, Plaza de los Caídos 1-5, 37008 Salamanca, Spain; 3 Departamento de Ingeniería Química, Facultad de Ingeniería Química, Universidad Nacional del Litoral, CP 3000, Santa Fe, Argentina

**Keywords:** essential oil, antibacterial activity, *Schinus molle* L., agar diffusion, effect of vapor method of cylindrical cavities, MIC, terpinenediols, 7-formyloxysabinen-2-ol

## Abstract

*Schinus molle* L. (aguaribay, aroeira-falsa, “molle”, family Anacardiaceae), a native of South America, produces an active antibacterial essential oil extracted from the leaves and fruits. This work reports a complete study of its chemical composition and determines the antibacterial activity of *Schinus molle* L. essential oil and its main components. The results showed that the crude extract essential oil has a potent antibacterial effect on *Staphylococcus aureus* ATCC 25923, a strong/moderate effect on *Escherichia coli* ATCC 25922 and moderate/weak one on *Pseudomonas aeruginosa* ATCC 27853.

## 1. Introduction

Essential oils are natural and complex volatile compounds [1,2], characterized by a strong odor, generally soluble in organic solvents with lower density than water [3,4]. They are produced in aromatic and medicinal plants as secondary metabolites and are obtained from various plant parts (flower, seed, leaves, twigs, bark, herbs, wood, fruits and roots) [3] and stored in secretory cells, cavities, vessels, or epidermal cells called glandular trichomes [4]. The family Anacardiaceae of pantropical occurrence includes a few representatives in temperate regions. The family comprises about 70 genera and 600 species. They are used traditionally as a healing, stomachic and antidiarrheal agent, due to the presence of tannins and oil resins [5]. In this family, *Schinus molle* L. (also known as California pepper and pink pepper) ERA 4350 (1996) was introduced from South America to most tropical and subtropical areas of the world, as well as the Mediterranean [6]. The “molle” was the sacred tree of the Incas, who were doing the planting and watering on the outlines of their palaces, temples and public building. From the resin exuded from the trunk a liquid rubber that served to embalm was obtained. There are several reports on the insecticidal [7] and antimosquito [8] activity of essential oils of *S. molle*. About 60% of essential oils have antifungal activity and 35% have antibacterial properties [9].

The chemical composition of the essential oil consists mainly of monoterpene hydrocarbons (e.g., α-pinene, β-pinene, sabinene, terpinen-4-ol…), and some sesquiterpenes such as (+) spathulenol and germacrene-D [10]. Essential oils act against microorganisms often causing instability in the plasma membrane leading to cell lysis. Although the antimicrobial activity can be triggered by a single chemical compound, it usually appears to result from a synergy between several chemicals in the oil [11].

The inherent activity of an oil may be related to its chemical composition, the proportions of the components and the interactions between them. Some studies have concluded that the oils as a whole have much greater antibacterial activity than a mixture of the major components, which suggests that minor components are critical for this activity and may have a synergistic effect or potentiating influence [12]. In this work we determined the antibacterial activity of the crude oil extract of *S. molle* (extracted from the ripe fruits and leaves of the plant) and its components against *Staphylococcus aureus* (ATCC 25923), representative of human superficial flora, *Escherichia coli* (ATCC 25922) representative of the intestinal flora and *Pseudomonas aeruginosa* (ATCC 27853) an ubiquitous bacterium representative of the environmental flora. We chose the agar diffusion method with cylindrical cavities for the determination of the inhibition halos (adapted from Clinical and Laboratory Standards Institute method M02–A10) [13,14], the test strips (agar diffusion) to determine the effect of vapor (volatility) [10] and the macrodilution method to determine the macrodilution Minimum Inhibitory Concentration (MIC) and Minimum Bactericidal Concentration (MBC) (an adaptation of method M07–A08, CLSI) [13]. 

## 2. Results and Discussion

### 2.1. Composition of the Essential Oil of Schinus Molle L.

The essential oils of the ground fruits and leaves were obtained by stream stripping. The essential oil analysis were performed by GC and GC-MS, and Table 1 shows the products quantified and identified using the NIST and Wiley databases. 

**Table 1:** Components and quantification of the essential oils of fruits and leaves of *S. molle*.

Number	R_t_	M	Compounds	%
1	8.51	136.2	thujene	1.48
2	9.14	136.2	α-pinene	5.32
3	11.47	136.2	sabinene	34.77
4	11.54	136.2	β-pinene	4.50
5	12.42	136.2	myrcene	1.72
6	14.17	136.2	α-terpinene	1.34
7	14.50	134.2	p-cymene	1.46
8	15.10	136.2	L-limonene	4.18
9	17.09	136.2	γ-terpinene	2.39
10	17.58	154.0	sabinene hydrate	0.16
11	18.54	136.2	α-terpinolene	0.51
12	20.06	154.0	NI (alcohol)	0.42
13	25.53	154.2	terpinen-4-ol	5.50
14	26.52	154.0	α-terpineol	0.62
15	38.59	204.4	α-copaene	0.37
16	40.06	204.0	β-elemene	1.97
17	41.53	204.3	β-caryophyllene	3.84
18	44.07	204.1	α-humulene	0.52
19	44.23	204.3	alloaromadendrene	0.85
20	46.00	204.3	germacrene-D	7.06
21	46.19	204.3	β-selinene	0.54
22	46.51	204.3	germacrene-B	3.87
23	47.07	204.3	α-muurolene	0.35
24	47.29	204.1	germacrene-A	0.60
25	47.59	204.2	γ-cadinene	1.22
26	48.23	204.3	δ-cadinene	1.14
27	51.57	220.1	(+)-spathulenol	3.91
28	52.05	220.1	caryophyllene oxide	1.02
29	55.55	222.0	δ-cadinol	2.11

**Figure 1:**
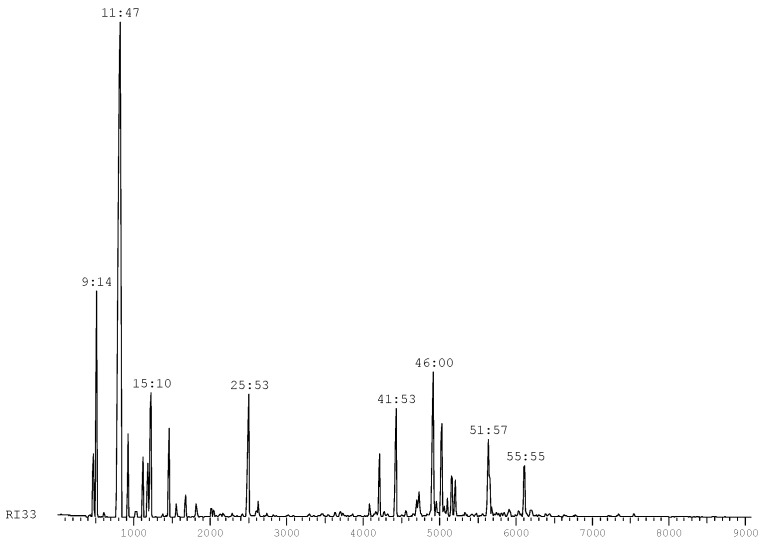
Chromatographic profile of *S. molle*.

The chromatographic profile of this essential oil (Figure 1) is consistent with the ranges defined by standard IRAM (Argentina) [15] for the essential oil of the fruits and leaves of *Schinus molle* L. (Table 2).

The essential oil was chromatographed with a low-medium pressure pump system, using a silica-gel flash type column prepared in the laboratory. Fractions were collected with an automatic collector. The obtained fractions were analysed by ^1^H-, ^13^C-NMR, bidimensional 2D NMR and GC-MS to determine the components. In previous studies [16], several components of *Schinus molle* L. oil were identified, among which sabinene was the main component, followed by (α, β)-pinene and terpinen-4-ol, accounting for a total of 94.0% of the essential oil. In this work we isolated and identified more polar components of the essential oil, which correspond to terpinenediol derivatives and formyloxy derivatives of sabinene and terpinene. The fractions obtained in this work by column chromatography used for antibacterial testing (and their compositions) are summarized in Table 3.

**Table 2:** Composition of the IRAM standard (Argentina).

Compounds	Minimum (%)	Maximum (%)
α-pinene	1.5	12.5
β-pinene	2.5	20.0
sabinene	31.0	55.0
terpinen-4-ol	0.5	13.5
germacrene-D	5.2	10.0

**Table 3:** Composition of *S. molle* oil fractions tested for antibacterial activity.

	Composition (%)				
	α -Pinene	β -Pinene	Sabinene	(+) Spathulenol	Terpinen-4-ol
	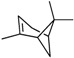	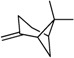	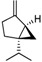	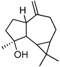	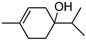
Fractions					
F01	19	2,5	25	-	-
F02	-	-	11	-	-
F03	-	-	12	16	-
F04	-	-	-	18	21
F05	-	-	-	14	25
F06	-	-	-	7	19

The most polar fraction F07 has as a major component a product with an M^+^ to 198, which corresponds to the molecular formula C_11_H_18_O_3_ (compound **1**). HMRSNa was measured as 221.1146 (calc. for the formula C_11_H_18_O_3_ 221.1148). The ^1^H-NMR spectrum shows a one H singlet at 8.11 ppm that can be attributed to a formyloxy group (HCOO-); at 4.18 ppm and 4.12 ppm there are two doublets of 1H each, *J* = 12.5 Hz, which can be attributed to the methylene group attached to the formyloxy group. At 0.96 ppm and 0.87 ppm two doublets of three H each (*J* = 7.0 Hz) are observed, corresponding to two methyl groups that can be attributed to an isopropyl group. The signals at 0.43 ppm (dd, *J* = 5.4 and 8.3 Hz) and 0.25 ppm (dd, *J* = 3.6 and 5.4 Hz) 1H each, can be attributed to a cyclopropane methylene group in a bicyclic system [5.3] like in the skeleton of sabinene [17]. The ^13^C-NMR spectrum confirms the skeleton carbons of sabinene, with the carbon of the methylene group of the cyclopropane ring at 13.0 ppm (Table 4). Thus, compound **1** corresponds to a new derivative of the sabinene skeleton.

**Table 4:** Attribution of the ^13^C-NMR data of compounds **1**–**3**.

Nº of C	δ ppm 1	Structure 1	δ ppm 2	Structure 2	δ ppm 3	Structure 3
1	32.2	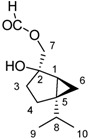	70.3	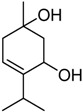	67.5	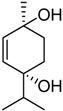
2	81.4		32.6		135.7	
3	32.7		119.8		133.7	
4	25.6		147.9		71.8	
5	34.7		71.9	**2**	27.3	**3**
6	13.0	**1**	24.6	Terpin-3-en-1,5-diol	33.7	Terpin-2-en-1,4-diol [18]
7	69.3	7-formyloxysabinen-2-ol	24.5		29.8	
8	30.5		34.7		37.6	
9	20.1		21.5		16.6	
10	20.1		21.5		17.8	
HCOO	161.3		--		--	

The major component of fraction F08 corresponds to a diol that shows in the mass spectrum a molecular ion at *m/z* 170, corresponding to a molecular formula C_10_H_18_O_2_. The ^1^H-NMR spectrum shows a multiplet integrating for 1H at 5.43 ppm, due to an olefinic hydrogen and at 3.80 ppm a 1H, m, signal of hydrogen atom geminal to an -OH group. Other signals are observed at 1.20 ppm (3H, s), corresponding to a methyl group geminal to a hydroxyl group (Me-7), at 1.03 ppm (3H, d, *J* = 7.1 Hz), for a methyl group at position 9 and at 1.00 ppm (3H, d, *J* = 7.1 Hz) corresponding to a methyl group at position 10. Taken together this data suggest a terpinenediol [18]. The ^13^C-NMR data of this new derivative **2** is given in Table 4 [19,20]. 

In fraction F09 another terpinene derivative was observed. The mass spectrum shows a molecular ion at 170 amu which corresponds to C_10_H_18_O_2_. Its spectroscopic data was identical to that described in the literature for compound **3** [21]. 

These formylated hydroxyl or hydroxylated derivatives are minor components that cannot be detected by GC or GC/MS. These products easily dehydrated at the high injector temperature giving rise to the corresponding hydrocarbons. No major influence of these derivatives has been observed in antibacterial activities studied.

### 2.2. Volatiles, Vapor Inhibition Halo Effects

Only the crude extract and fraction F01 showed vapor inhibition halos derived from the effect of essential oil of *S. molle*. The smallest inhibition halo fraction of F01 is due to the fact that this fraction is more diluted (with an adjusted concentration of 90 mg/mL), below the concentration of components in the crude extract. Analysis of the results led us to conclude that the effect of vapor is mainly due to the presence of low molecular weight monoterpenes, namely (α, β)-pinene (Table 5).

**Table 5:** Inhibition halos (mm) resulting from tests of vapor diffusion in agar.

Strains	Arithmetic means of the inhibition halos (mm) *		
	Crude extract	F01	F02, F03, F04, F05 and F06
*S. aureus* ATCC 25923	32.7 ± 1.1	14.6 ± 0.7	0.0
*E. coli* ATCC 25922	0.0	0.0	0.0
*P. aeruginosa* ATCC 27853	0.0	0.0	0.0

* Essays in hexaplicate.

### 2.3. Crude Extract—Cylindrical Cavities Agar Diffusion Method

After analyzing the oil volatiles, analytical tests by the agar diffusion method in cylindrical cavities were done, whose results are summarized in Table 6. From the results, we conclude that the oil of *S. molle* has the highest inhibitory effect against *S. aureus* ATCC 25923 strains, which showed resistance only to the 1:32 dilution. For *E. coli* ATCC 25922 the inhibitory effect is smaller but still (according to the criteria defined in 3.1) only has a strong inhibitory effect for the crude extract (undiluted). *P. aeruginosa* ATCC 27853 strains are less sensitive, with a maximum range of moderately sensitive, for this oil. We carried out the test with an intermediate dilution 1:10.5 (90 mg/mL) in order to carry out a comparative analysis of the fractions obtained by column chromatography and described in Table 7, Table 8 and Table 9.

**Table 6:** Comparison between crude extract and its dilutions. (Tukey’s test at 5% probability).

Oil of *S. molle*			Inhibition halos (mm) ± standard deviation *			
Dilution	Concentration (mg/mL)	*S. aureus*		*E. coli*	*P. aeruginosa*	
Crude	950 **	38.2 ± 0.2 Aa		22.0 ± 0.2 Ba	16.0 ± 0.2 Ca	
1:2	475	29.2 ± 0.2 Ab		15.3 ± 0.2 Bb	11.5 ± 0.2 Cb	
1:4	237	25.3 ± 0.2 Ac		10.8 ± 0.2 Bc	7.2 ± 0.2 Cc	
1:8	118	17.5 ± 0.2 Ad		8.7 ± 0.2 Bd	5.3 ± 0.2 Cd	
1:10,5	90	15.3 ± 0.2 Ad		5.7 ± 0.2 Bd	5.0 ± 0.2 Cd	
1:16	60	14.5 ± 0.2 Ad		5.0 ± 0.2 Bd	5.0 ± 0.2 Cd	
1:32	30	5.3 ± 0.2 Ae		5.0 ± 0.2 Bd	5.0 ± 0.2 Cd	
Positive control		40.5 ± 0.1		38.2 ± 0.7	39.2 ± 0.8	
Negative control		5.0		5.0	5.0	

* The value of 5.0 mm corresponds to the diameter of the cylindrical cavity, no inhibition zone; ** The calculation of the concentration was based on the density of crude oil extract of *S. molle* (d = 0.95). Capital letters in the same line indicate no statistical difference, the same lowercase letters in the same column indicate no statistical difference (Tukey’s HSD test, *p* > 0.05).

**Table 7:** Results expressed as mean diameters of the inhibition halos in millimeters (mm) ± standard deviation (Tukey’s test at 5% probability).

Organism	*S. molle* Fractions	Average of the halos of inhibition in millimeters (mm) ± SD				
		90 mg/mL	45 mg/mL	22.5 mg/mL		
*S. aureus*	F01	29.5 ± 0.3	25.8 ± 0.3	17.7 ± 0.3	a *	
	F02	22.7 ± 0.3	18.5 ± 0.3	14.6 ± 0.3	c	
	F03	25.5 ± 0.3	14.7 ± 0.3	11.3 ± 0.3	b	
	F04	14.3 ± 0.3	11.5 ± 0.3	8.2 ± 0.3	d	
	F05	22.3 ± 0.3	16.0 ± 0.3	12.7 ± 0.3	c	
	F06	22.3 ± 0.3	16.0 ± 0.3	12.5 ± 0.3	c	
*E. coli*	F01	11.0 ± 0.2	6.8 ± 0.2	5.0 ± 0.2	c *	
	F02	11.3 ± 0.2	5.7 ± 0.2	5.0 ± 0.2	c	
	F03	5.0 ± 0.2	5.0 ± 0.2	5.0 ± 0.2	d	
	F04	10.8 ± 0.2	6.5 ± 0.2	5.0 ± 0.2	c	
	F05	18.5 ± 0.2	13.5 ± 0.2	6.8 ± 0.2	a	
	F06	16.3 ± 0.2	10.0 ± 0.2	9.8 ± 0.2	b	
*P. aeruginosa*	F01	8.7 ± 0.2	6.8 ± 0.2	5.5 ± 0.2	b *	
	F02	11.3 ± 0.2	6.2 ± 0.2	5.2 ± 0.2	a	
	F03	10.2 ± 0.2	7.3 ± 0.2	5.7 ± 0.2	a	
	F04	8.0 ± 0.2	6.0 ± 0.2	5.7 ± 0.2	b	
	F05	9.3 ± 0.2	7.3 ± 0.2	6.8 ± 0.2	a	
	F06	9.0 ± 0.2	8.0 ± 0.2	6.5 ± 0.2	a	

* Same letters in a column indicate no statistical difference (Tukey’s HSD test, *p* > 0.05).

**Table 8:** Experimental CIM and CMB results.

Oil of *S. molle*		*S. aureus*		*E. coli*		*P. aeruginosa*	
Final dilutions *	Concentrations *	RZ	MBC	RZ	MBC	RZ	MBC
	(mg/mL)	(Cor)	(CFU/mL)	(Cor)	(CFU/mL)	(Cor)	(CFU/mL)
1:4	22,50	AV	<1	AV	<1	AV	<1
1:8	11,25	AV	<1	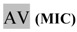	<1	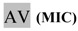	=440
1:16	5,63	AV	<1	Rv	>3000	Rv	>3000
1:32	2,81	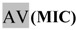	<1	Rv	>3000	Rv	>3000
1:64	1,41	Rv	>3000	Rv	>3000	Rv	>3000
1:128	0,70	Rv	>3000	Rv	>3000	Rv	>3000
C− (negative/sterility)		AV	<1	AV	< 1	AV	<1
C+ (positive/growth)		Rv	>3000	Rv	>3000	Rv	>3000

* Dilutions and final concentrations, taking into account the volume of inoculum added. **MIC**: Minimum Inhibitory Concentration; **MBC**: Minimum Bactericidal Concentration; **RZ**: resazurin test; **AV**: blue/violet tone; **Rv**: pink/red tone; **NR**: test not performed. **CFU**: colony forming units.

**Table 9:** Shows the inhibitory effects greater and lower in oil components.

		*S. aureus*	*E. coli*	*P. aeruginosa*
EB	MIC (mg/mL)	2.81	11.25	11.25
	DAC (mm)	38.2 ± 0.2	22.0 ± 0.2	16.0 ± 0.2
DAV (mm)	EB	32.7 ± 1.1	0.0	0.0
	F01	14.6 ± 0.7	0.0	0.0
Greater inhibitory effect	Fractions	F01	F05	F02
	Components	Pinenes/Sabinene	Terpinen-4-ol	Sabinene
	Halos (mm)	29.5 ± 0.3	18.5 ± 0.2	11.3 ± 0.2
Lower inhibitory effect	Components	(+)Spathulenol	(+)Spathulenol	(+)Spathulenol

**MIC**: Minimum Inhibitory Concentration; **DAV**: vapor diffusion method on agar; **DAC**: cylindrical cavities agar diffusion method; **EB**: extract oil at a concentration of 90 mg/mL.

### 2.4. Fractions of the Oil of S. molle L.—Cylindrical Cavities Agar Diffusion Method

To analyze of each of the fractions obtained by CC, three different concentrations (90 mg/mL, 45 mg/mL and 22.5 mg/mL) were prepared for each strain (Table 7, Table 8 and Table 9). Analyzing these results, we conclude (as for crude extract) that the inhibitory effect of each fraction decreases with increasing dilution, demonstrating direct proportionality between the total concentrations of components and increased inhibition zone. All fractions have a greater inhibitory effect for the highest concentration (90 mg/mL), with the halo of inhibition (29.5 ± 0.3 mm) caused by fraction F01 (19% α-pinene, 2.5% β-pinene and 25% sabinene) against the Gram-positive strains standing out. Comparing this result with the halo of inhibition obtained by the effect of the vapor (14.6 ± 0.7 mm) it follows that there may be a synergy between these components (pinenes and sabinene) when they act against *S. aureus* ATCC 25923. In future studies would be interesting to separate available fractions with pinenes and sabinene isolated in equal concentration, in order to reliably conclude which of the two components has higher antibacterial activity against *S. aureus* ATCC 25923 and if indeed there is synergy between them. For the lowest concentration of 22.5 mg/mL the Gram-positive strain did not exceeded a value of 17.7 ± 0.3 mm for the halo of inhibition (moderately susceptible) while the remaining strains, *E. coli* ATCC 25922 and *P. aeruginosa* ATCC 27853, were resistant to oil at this concentration.

For *S. aureus* ATCC 25923 and comparing fractions F03 and F02, it was found that the inhibition zone is slightly higher than for F03, probably due to the increased concentration of sabinene and not the presence of (+)-spathulenol so there appears to be antagonism between these two components. Comparing F04, F05 and F06, it is notable that the fraction containing the highest percentage of (+)-spathulenol showed the lowest activity against the Gram-positive strain, possibly due to an antagonism between (+)-spathulenol and terpinen-4-ol. *E. coli* ATCC 25922 is more sensitive to terpinen-4-ol. F05 (14% (+)-spathulenol and 25% terpienen-4-ol) is the fraction with the highest inhibitory effect in this strain, followed by F06 (7% (+)-spathulenol and 19% terpinen-4-ol). Comparing the results, we highlight the possible effect of the antagonistic (+)-spathulenol on terpinen-4-ol and sabinene in both Gram-negative strains. Comparing the fractions F01, F02 and the absence of inhibition zone by the effect of vapor in Gram-negative strains, we conclude that this may also be associated with an antagonism between the pinenes and sabinene at this group. For *P. aeruginosa* ATCC 27853, the fraction that showed highest rate of inhibition was F02 (11% sabinene) followed by F03 (12% sabinene and 16% (+)-spathulenol). On the contrary, *E. coli* ATCC 25922 showed better inhibition halos for the terpinen-4-ol, possibly due to the greater symmetry of the biphospholipidic outer membrane characteristic of the genus *Pseudomonas* spp, compared to the family Enterobacteriaceae.

### 2.5. Method of Macrodilution—MIC and MBC

According to the methodology described in 2.9, we obtained the values summarized in Table 8. Given the difficulty in visualizing the turbidity in the tubes due to the oil emulsion, the MIC results were evaluated according to the resazurin test. Predictably, and in accordance with the agar diffusion test, the oil has a stronger inhibitory effect on the Gram-positive strain with a MIC of 2.81 mg/mL corresponding to the dilution 1:32. The actual value for the MIC and MBC will be between 1.41 mg/mL and 2.81 mg/mL. For Gram-negative strains, the MIC and MBC observed were equal (1:8 dilution, 11.25 mg/mL) with the actual concentrations varying between 5.63 mg/mL and 11.25 mg/mL. Although a count of 440 CFU/mL was obtained for *P. aeruginosa* ATCC 27853, this value is less than 0.1% (1,000 CFU/mL) of the original inoculum (10^6^ CFU/mL), strengthening the lower bacterial effect (due to the proximity of MIC values and MBC) of oil of *S. molle* on this strain. Comparing the macrodilution and cylindrical cavities agar diffusion methods, there appears to be a good correlation between MIC values and inhibition halos for the lower concentrations, possibly due to several factors, such as the fact of being an oil (lipophilic), in aqueous emulsion it may not occur an efficient contact between the oil and the bacterial cell and poor diffusion in the agar.

Despite the low antibacterial activity, sabinene showed the best inhibitory effect on *P. aeruginosa* ATCC 27853, indicating that this strain is very insensitive/resistant to this oil, possibly due to low outer membrane permeability of strongly/moderately hydrophobic compounds and the multidrug efflux systems characteristic of this kind of organism. (+)-Spathulenol is the component that has a lower activity, or lack of it, with possible antagonism with the terpinen-4-ol level in all tested strains and the sabinene only in Gram-negative strains, Table 9.

## 3. Experimental

### 3.1. Plant Material and Essential Oil Extraction

The essential oil of the fruits and leaves of *Schinus molle* L. studied in this paper came from specimens of aguaribay (“molle”) grown in the “Angel Gallardo” Experimental Operation Center of the School of Chemical Engineering at the Universidad Nacional del Litoral, Ministerio de la Producción de la Provincia de Santa Fé, Santa Fé, Argentina, located at 31° 32' 55'' S and 60° 41' 27'' O. Final identification of *Schinus molle* L. was made by Juan de Dios Muñoz and the specimen is deposited in the Herbarium ERA 4350 (1996)-Jardín Botánico Oro Verde, Facultad de Ciencias Agrarias, UNER - Ruta provincial 11, km 10,5, Oro Verde, Entre Ríos, Argentina [22]. 

The oil extraction was performed on the crushed leaves and ripe fruits (industrial extraction) by steam distillation using percolation. In this technique the plant material is immersed in the mass of water, which is heated and generates saturated steam in contact with the mass of vegetable oil-bearing particles, physically removing it, and this mixture is then condensed and decanted, to separate the essential oil layer from the aqueous layer.

### 3.2. Separation of Oil Components

The overall analysis was performed by GC/MS on a Shimazdu QP-5000 gas chromatograph. The separation of oil components was performed by column chromatography on flash silica, using as eluent hexane and mixtures of hexane/EtOAc of increasing polarity. The fractions were analyzed by TLC. The compounds were identified by spectroscopic methods such as ^1^H-, ^13^C-NMR, 2D NMR, GC/MS and High Resolution Mass Spectrometry. 

### 3.3. Bacterial Strains

The strains tested were: *Staphylococcus aureus* (ATCC 25923), *Escherichia coli* (ATCC 25922) and *Pseudomonas aeruginosa* (ATCC 27853) from the American Type Culture Collection (ATCC, LGC Standards S.L.U., Barcelona, Spain). The criterion for selection of these strains was in accordance with the standard CLSI M26-A [14].

### 3.4. Culture Media and Solutions

To determine the antibacterial activity, we used the following resources and solutions: pre-prepared commercial cards of Muller-Hinton Agar (MH2, Ref 43301, BioMérieux SA, Linda-a-Velha, Lisboa, Portugal) with 4 mm thick broth Muller-Hinton Broth (MHB, Ref 724245, Oxoid Ltd, Hampshire, England), aqueous solvent for the oil emulsion (0.15% agar + 5% DMSO) [16] and 1% resazurin solution in saline (NaCl 0.85%) to colorimetrically detect bacterial growth.

### 3.5. Bacterial Cultures

For reconstitution of lyophilized bacterial strains in the swabs provided (Culti-Loops, Oxoid Ltd, Hampshire, England), we proceeded as follows: the swab was allowed to reach room temperature, the solid MH2 medium was inoculated by passing the swab on the agar and incubated 18 h–24 h/37 ± 0.5 °C. A second plate was inoculated by streaking from an isolated colony and incubated at the same temperature for 18 h–24 h. Thus we obtain the primary culture (stock culture). The working cultured is obtained by subculturing the primary culture in the same way and under the same incubation conditions [23].

### 3.6. Standardization of Inoculum

All inocula were prepared and standardized from the working culture in accordance with the methodology proposed by the CLSI [24]. A sterile loop was removed from 1 to 2 colonies, emulsified in a sterile solution of NaCl 0.85% (Ref 20040, BioMérieux S.A., Linda-a-Velha, Lisboa, Portugal) and read on a DENSIMAT densitometer (BioMérieux S.A., Linda-a-Velha, Lisboa, Portugal) to ensure a microbial load of approximately of 10^8^ CFU/mL (turbidity corresponding to McFarland standard 0.5). Followed by addition of 0.1 mL of standardized inoculum and 9.9 mL of sterile MHB broth, resulting in a microbial load of approximately 10^6^ CFU/mL, the latter being the solution inoculated to perform all tests. Confirmation of the inoculum was made by successive dilutions of the working solution up to 10^3^ CFU/mL and 0.1 mL of further spread in the MH2 medium. The obtention of approximately 100 CFU/mL confirms the microbial load of the original inoculum.

### 3.7. Method of the Effect of Vapor—Diffusion in Agar

To study the antibacterial activity due to volatility (vapor effects) of the crude oil and fractions of *S. molle*, we used the method described by Lysine *et al.* [10]. The crude oil and corresponding fractions were introduced in the swab inoculum (10^6^ CFU/mL) and pressed into the wall of the tube to remove excess liquid. It then spread evenly with the swab, the inoculum on the agar plate MH2 and left to stand for 15 min so they absorb the culture medium around the inoculum. In order to do a comparative test with the agar diffusion method using the technique of cylindrical cavities, it was added the same amount, 70 µL of the sample in the centre of the lid of the Petri dish, at a distance of approximately 1 cm from the agar inoculated, inverted the plate and incubated during 18 h–24 h/37 ± 0.5 °C. Assays were performed in hexaplicate.

### 3.8. Method of Diffusion in Cylindrical Agar Cavities

The antimicrobial activity was evaluated according to the agar diffusion method proposed by the Clinical and Laboratory Standard Institute (CLSI, Wayne, Pennsylvania, PA, USA), but replacing the sterile paper discs impregnated with the sample by cylindrical cavities of 4 mm in height and 5 mm in diameter, punched into agar with a thickness of 4 mm [24,25]. As a positive control, we used the antibiotic gentamicin (Sigma-Aldrich, Sintra, Portugal) at a concentration of 10 mg/mL as negative control and the dilution solution (aqueous solution agar 0.15% + 5% DMSO). The cylindrical cavities were filled with 70 mL of oil in different concentration and their controls, after a rest period of 15 min at room temperature, which allows the diffusion of the components, the plates were incubated for 18 h–24 h/37 ± 0.5 °C. After the incubation period, the inhibition halos were measured by visual observation and a stereoscopic microscope. All assays were performed in hexaplicate. 

### 3.9. Macrodilution Broth

To determine the MIC and MBC the technique of macrodilution in MHB broth was used, according to the methodology of CLSI M07–A8 [13]. This approach was complemented by the colorimetric resazurin test for improved detection of bacterial growth [26]. The resazurin was added only at the end of the incubation period. Previously, the inoculum of the microorganism to be tested was prepared at a concentration of 10^6^ CFU/mL in MHB (standardized to 3.6), the solution of the crude extract of *S. molle* was emulsified in the dilution solution to obtain a concentration of 90 mg/mL and finally the solution of resazurin was sterilized through a 0.22 µm porosity filter (02 Filtropur S, Sarstedt, Germany). In eight sterile tubes containing 1 mL of each MHB broth in double concentration, 1 mL of essential oil at a concentration of 90 mg/mL was added to the first tube and homogenized. Then 1 mL was withdrawn from the first tube and transferred to the second tube and so on until the last dilution. In the end, 1 mL of inoculum was added to all tubes (final concentration of oil is equivalent to 1/3) except for the negative control. As positive control we used MHB broth (double concentration) with 1 mL of inoculum. As a negative control, we used 1 mL of MHB broth (double concentration) with 1 mL of the crude extract oil. All test tubes, mixed thoroughly, were incubated for 18 h–24 h/37 ± 0.5 °C. After incubation, the tubes were visually examined and tested with resazurin, adding 9 mL of resazurin to 1 mL of sample. For the determination of the MBC, samples were inoculated by spreading 0.1 mL of each tube (including controls) on MH2 agar and incubating during 18 h–24 h/37 ± 0.5 °C and then proceeding to count the colony forming units (CFU). All assays were performed in triplicate. The tube with the lowest concentration of oil discolored from resazurin, was considered as corresponding to the MIC. The MBC corresponds to a dilution that shows inhibition of bacterial growth of at least 99.9% of the original inoculum (106 CFU/mL) [14]. 

### 3.10. Inhibition Halos

The values of the inhibition halos in relation to the agar diffusion methods were measured in mm and include 5 mm diameter cylindrical cavity in accordance with the standard M100–S19, CLSI [27]. The scale of measurement (cavity diameter included) was as follows: ≥20 mm strong inhibitory effects, <20 to 12 mm moderate inhibitory effects, >12 mm weak inhibitory effects. Assays were performed in hexaplicate and data presented as mean ± standard deviation. Readings were made with a ruler and confirmed with a stereoscopic microscope.

### 3.11. Statistical Analysis

Data analysis was performed using the SPSS 17.0 statistics program, by analysis of variance, using two-way ANOVA and comparison of means by a study comparing a posteriori (post hoc)—Tukey Test—allowed to differentiate minimum between means when the probability value *p* < 0.05.

## 4. Conclusions

In general terms and in accordance with the results obtained it is concluded that the oil of *Schinus molle* has the greatest effect on the bacterial strain *S. aureus* ATCC 25923, strong/moderate on *E. coli* ATCC 25922 and moderate/poor on *P. aeruginosa* ATCC 27853. The greatest antimicrobial effect (with a possible synergy between them) in the Gram-positive strain arises mainly from the presence of monoterpene hydrocarbon phytochemical such as the pinenes and sabinene and of these only the pinenes (due to their volatility) have an inhibitory effect through the vapor. Of the four monoterpenes tested terpinen-4-ol was shown to have lower activity in Gram-positive strains. The terpinen-4-ol, which had the best antibacterial activity had an inhibitory effect with strong/moderate in *E. coli* ATCC 25922, possibly due to its lower molecular weight (compared to (+)-spathulenol), thus allowing their passage through the hydrophobic pathways across the outer membrane or ability to distribute the cell membrane, increasing permeability and destabilizing the cell structure. This work demonstrated the strong potential of this bacterial strain oil in Gram-positive *S. aureus* ATCC 25923 and less effect on the Gram-negative strain tested. Thus, essential oils can be a bridge between traditional medicine and modern medicine [5]. Apart from the biological activity studies, the isolation and structure determination or two new terpene derivatives is described for the first time in this manuscript. 
